# American College of Surgeons Operative Standards and Breast Cancer Outcomes

**DOI:** 10.1001/jamanetworkopen.2024.46345

**Published:** 2024-11-20

**Authors:** Crystal D. Taylor, Ton Wang, Alison S. Baskin, Brandy Sinco, Tasha M. Hughes, Daniel J. Boffa, Judy C. Boughey, Lesly A. Dossett

**Affiliations:** 1 Department of Surgery, Michigan Medicine, Ann Arbor; 2 Center for Health Outcomes and Policy, Michigan Medicine, Ann Arbor; 3 Institute for Healthcare Policy and Innovation, University of Michigan, Ann Arbor; 4 National Clinician Scholars Program, University of Michigan, Ann Arbor; 5 Department of Surgery, Duke University, Durham, North Carolina; 6 Department of Surgery, University of California, San Francisco; 7 Department of Surgery, Section of Thoracic Surgery, Yale University School of Medicine, New Haven, Connecticut; 8 American College of Surgeons Cancer Research Program, Chicago, Illinois; 9 Division of Breast and Melanoma Surgical Oncology, Department of Surgery, Mayo Clinic, Rochester, New York

## Abstract

**Importance:**

The American College of Surgeons (ACS) operative standards were established to detail critical elements of cancer surgery, reduce technical variation, and improve outcomes. Two of the 6 operative standards target adequate axillary surgery for breast cancer. The potential association of the operative standards with short-term oncologic outcomes, such as nodal yield and nodal positivity rates, is currently unknown.

**Objective:**

To evaluate the potential association of the ACS operative standards with short-term oncologic outcomes in breast cancer.

**Design, Setting, and Participants:**

A cohort study was performed using data on 1 201 317 women 18 years or older who underwent sentinel lymph node biopsy (SLNB) or axillary lymph node dissection (ALND) for invasive breast cancer from January 1, 2012, to December 31, 2020. Patients were identified using the National Cancer Database (NCDB), a clinical oncology database encompassing approximately 70% of new cancer diagnoses, sourced from hospital registry data from 1317 facilities. Statistical analysis was performed from October 2023 to June 2024.

**Exposure:**

Sentinel lymph node biopsy or ALND.

**Main Outcomes and Measures:**

Reliability-adjusted facility-level lymph node yield and nodal positivity rate for each procedure were calculated using generalized linear mixed models, Poisson regression, and logistic regression with facility-level random intercepts.

**Results:**

The cohort included 1 201 317 women with a median age of 62 years (IQR, 53-70 years). Facility-level nodal yield ranged from 1 to 6 for SLNB and from 6 to 22 for ALND. Median facility-level nodal yield for SLNB was 2.6 (IQR, 2.3-3.0) and the nodal positivity rate for SLNB was 12.2% (IQR, 11.0%-13.7%), with rates ranging from 6% to 21%. A weak correlation between facility-level lymph node yield and nodal positivity was observed (Spearman correlation coefficient, 0.17). Median nodal upstaging rate (≥4 positive nodes) for ALND was 30.5% (IQR, 26.5%-35.0%), with rates ranging from 11% to 54%; median nodal yield was 12.2 (IQR, 10.9-13.6). A strong correlation between nodal yield and nodal upstaging rates was observed (Spearman correlation coefficient, 0.53).

**Conclusions and Relevance:**

In this cohort study of women undergoing axillary surgery for invasive breast cancer, facility-level variation in lymph node yield was present for both SLNB and ALND, which could potentially be improved through the ACS operative standards. However, this variation had mixed associations with nodal positivity and upstaging rates, suggesting the association of the ACS operative standards with oncologic outcomes may be mixed.

## Key Points

**Question:**

What potential association could the American College of Surgeons (ACS) operative standards for cancer surgery have with short-term oncologic outcomes in breast cancer?

**Findings:**

In this cohort study of 1 201 317 women identified using the National Cancer Database, facility-level median nodal yield for sentinel lymph node biopsy was 2.6 (IQR, 2.3-3.0) and facility-level median nodal positivity rate was 12.2% (IQR, 11.0%-13.7%). Facility-level median nodal yield for axillary lymph node dissection was 12.2 (IQR, 10.9-13.6) and facility-level median nodal upstaging rate was 30.5% (IQR, 26.5%-35.0%).

**Meaning:**

This study’s findings suggest the ACS operative standards could have mixed associations with short-term oncologic outcomes in breast cancer.

## Teaser

This cohort study evaluates the potential association of the American College of Surgeons operative standards for cancer surgery with short-term oncologic outcomes in breast cancer.

## Introduction

Although many cancer quality improvement efforts have focused on measurement and reporting of the diagnostic, staging, and adjuvant therapy processes, relatively little attention has been paid to the measurement and reporting of potential differences in surgical technical quality.^1^ To address this gap, the American College of Surgeons (ACS) recently published 134 operative standards across 15 cancer types that identify key steps during optimal cancer surgery that can be leveraged as a quality assurance tool for practicing surgeons.^1^ The Commission on Cancer (CoC), which accredits over 1500 hospitals nationwide and serves more than 70% of all patients with cancer in the US, implemented 6 standards in 2020.^1,2^ All CoC facilities are expected to reach 80% compliance by 2024.

Four of the 6 standards rely on reporting via synoptic operative reporting, a previously rarely used communication tool for surgeons. This effort overall represents an unprecedented attempt to standardize, report, and improve technical quality. Therefore, understanding whether the current slate of ACS operative standards improves outcomes is critical, as well-designed standards could reduce technical variation and improve surgical quality.^1^ Alternatively, ineffective standards would place costly and unnecessary administrative burdens on clinicians and CoC hospitals without directly benefiting patients. Although the current ACS operative standards focus on cancer surgery, if this approach is proven effective, there would be substantial rationale for significant expansion to all surgical disciplines.

Of the 6 implemented operative standards, 2 (standards 5.3 and 5.4) target axillary surgery for breast cancer.^3^ Specifically, these 2 standards outline the critical components of performing a sentinel lymph node biopsy (SLNB) and axillary lymph node dissection (ALND). These standards were developed based on expert opinion and retrospective data demonstrating that patients with breast cancer undergoing lymph node sampling meeting certain nodal yields have improved overall survival compared with those who do not.^4,5,6,7,8,9^ High-quality axillary surgery is an important potential target for technical standardization because accurate staging informs adjuvant therapy recommendations. For example, the decision to administer postmastectomy radiotherapy, adjuvant targeted therapies such as cyclin-dependent kinase 4/6 inhibitors, and anthracycline-containing chemotherapy regimens are all influenced by the pathologic nodal stage.^10,11^ As more effective adjuvant therapies have contributed to improvements in breast cancer survival,^12,13^ careful assessment of the accuracy of lymph node staging, typically improved with lymph node yield, is needed.

Understanding the potential association of the ACS operative standards with oncologic outcomes requires a baseline assessment of current levels of variation that could be targeted for improvement. Although several studies have explored variation in lymph node yield at the patient level, operative standards aim to target surgeon behavior at the facility level. The level of potential variation in the quality of axillary surgery by facility or surgeon is unknown. On the one hand, many surgeons and facilities may already perform these procedures with high technical quality, leaving little room for improvement. On the other hand, ALND is a low-volume procedure for many surgeons and facilities, and an association between volume and quality may exist. For example, the National Comprehensive Cancer Network (NCCN) recommends examination of at least 10 lymph nodes during level I and level II ALND for pathologic evaluation to accurately stage the axilla.^11^ Thus, the objective of this study is to evaluate the potential association of the current ACS operative standards in breast cancer with oncologic outcomes by examining facility-level variations in nodal yield and the association this variation has with the nodal stage before implementation of the ACS standards. We describe variation in lymph node sampling, identify areas where the association of operative standards with oncologic outcomes may be limited by already high performance (ie, ceiling effect), and identify areas where technical improvements related to operative standards are most likely to have an association with oncologic outcomes.

## Methods

### Study Design

We conducted a cohort study of patients in the National Cancer Database (NCDB) from January 1, 2012, to December 31, 2020. This study was deemed exempt by the University of Michigan institutional review board due to the use of deidentified data, and patient informed consent was waived. This report follows the Strengthening the Reporting of Observational Studies in Epidemiology (STROBE) reporting guideline for cohort studies.^14^

### Data Source

The NCDB is a clinical oncology database sourced from hospital registry data from more than 1500 CoC-accredited facilities.^15^ With over 34 million records, it is the largest clinical cancer registry in the world, capturing approximately 70% of new cancer diagnoses in the US.^2,16^

### Study Population

Our cohort included women aged 18 years or older undergoing axillary surgery (≥1 lymph node examined) for invasive breast cancer. Women were excluded if they had stage IV breast cancer, received treatment outside of their reporting facility, received neoadjuvant chemotherapy, or had clinical T0 disease or pathologic T0 disease. Only facilities that performed a combined total of at least 10 axillary surgical procedures per year were included to obtain an accurate estimate of facility performance. Hospital volume was based on the mean annual breast cancer case count and categorized as low (10-99 cases), medium (100-199 cases), or high (≥200 cases). These definitions were based on previously published ranges and designed to ensure there was an adequate number of facility-level and patient-level data across groups for multilevel analysis.^17,18^

### Exposure

The NCDB did not include a dedicated variable for the SLNB examination until 2018. In 2018, the *SLNExam* variable became available in the NCDB and was used to evaluate patients who underwent SLNB alone from 2018 to 2020 and to quantify the number of lymph nodes examined during SLNB. Patients undergoing SLNB alone before 2018 were not included in the analysis. Using the *RXSummScopeRegLN2012* variable, we classified women as having undergone ALND or completion ALND after SLNB from 2012 to 2020. We then used the *RegionalNodesExamined* variable to quantify the number of lymph nodes removed. Although the NCDB includes lymph node values up to 90 or more, we truncated possible lymph nodes examined to 40 (values >40 were considered erroneous and excluded).

### Study Outcomes

Our primary outcomes were the mean, median, and range of lymph nodes examined, as well as the nodal positivity rate at each facility for patients undergoing SLNB and ALND (with or without SLNB). As a secondary outcome, we evaluated potential correlations between the number of lymph nodes examined and the nodal positivity rate for each axillary surgery by facility. We determined the percentage of facilities performing ALND that removed a mean of less than 10 lymph nodes. For SLNB, we evaluated the correlation between mean number of lymph nodes examined and nodal positivity rate (obtaining ≥1 positive nodes). For ALND, we further evaluated the correlation between mean lymph node yield and rates of obtaining 4 or more positive lymph nodes, as this would upstage patients from pathologic N1 to N2 disease and potentially change adjuvant therapy recommendations.

### Statistical Analysis

Statistical analysis was performed from October 2023 to June 2024. Reliability-adjusted estimates for count variables, such as lymph node yields, were generated from Poisson regression models with random intercepts for each hospital, also known as generalized linear mixed models (GLMMs). Similarly, the reliability-adjusted estimates for binary outcomes, such as 1 or more positive lymph nodes, were computed from GLMMs for logistic regression with random intercepts for hospitals. A GLMM with a hospital random intercept accounts for clustering within hospitals and is equivalent to an empirical bayesian estimate.^19^ The time series estimates were computed from GLMMs with a categorical indicator for the year and random intercepts for each hospital.

Key reliability-adjusted estimates for mean lymph node yields and nodal positivity are displayed by caterpillar plots. Reliability-adjusted estimates for mean lymph nodes examined and nodal positivity are displayed by scatterplots. The Spearman correlation coefficient was used to calculate the correlation between the mean number of lymph nodes examined and nodal positivity because Spearman correlation detects any increasing or decreasing trends, even if the trend is not linear.^20^ *P* < .05 was considered significant, and all *P* values were 2-sided. All statistical analyses were performed using SAS statistical software, version 9.4 (SAS Institute Inc).

## Results

### Patient Population

A total of 1 201 317 women (median age, 62 years [IQR, 53-70 years]) undergoing axillary surgery for breast cancer at 1317 CoC sites from 2012 to 2020 were eligible for analysis. Of these women, 915 380 (76.2%) underwent SLNB, 186 861 (15.6%) underwent SLNB with completion ALND, and 99 076 (8.2%) underwent ALND (Table 1). Patient distribution by facility characteristics is noted in Table 1. There were 782 990 patients (65.2%) treated at high-volume centers. The case volume for each axillary surgery is provided in eTables 1, 3, 4, and 5 in Supplement 1. The median annual volume of SLNB performed for facilities was 57.3 (IQR, 28.0-110.0). The median annual volume of ALND was 6.4 (IQR, 3.6-11.0). Between 2012 and 2020, the median number of ALND by facility decreased from 9.0 (IQR, 5.0-16.0) to 3.0 (IQR, 2.0-6.0). The median annual volume of SLNB with completion ALND was 10.7 (IQR, 5.7-20.3).

**Table 1.** Characteristics of Women Undergoing Axillary Surgery for Breast Cancer

Characteristic	Patients, No. (%) (N = 1 201 317)
Age, median (IQR), y	62 (53-70)
Clinical T category	
cT1	766 803 (63.8)
cT2	241 552 (20.1)
cT3	29 181 (2.4)
cT4	7984 (0.7)
cTis	125 716 (10.5)
Unknown	30 081 (2.5)
Clinical N category	
cN0	1 070 605 (89.1)
cN1	83 906 (7.0)
cN2	8366 (0.7)
cN3	4045 (0.3)
Unknown	34 395 (2.9)
Pathologic T category	
pT1	758 333 (63.1)
pT2	266 366 (22.2)
pT3	33 990 (2.8)
pT4	6093 (0.5)
pTis	100 640 (8.4)
Unknown	35 895 (3.0)
Pathologic N category	
pN0	914 427 (76.1)
pN1	198 724 (16.5)
pN2	37 459 (3.1)
pN3	16 201 (1.3)
Unknown	34 506 (2.9)
Axillary surgery	
SLNB	915 380 (76.2)
SLNB with completion ALND	186 861 (15.6)
ALND	99 076 (8.2)
Facility type^a^	
Community cancer	83 014 (6.9)
Comprehensive center	481 783 (40.1)
Academic center	345 001 (28.7)
Integrated network	246 513 (20.5)
Unknown	45 006 (3.7)
Annual case volume	
Low-volume facility (<100)	137 267 (11.4)
Medium-volume facility (100-199)	281 060 (23.4)
High-volume facility (≥200)	782 990 (65.2)

Abbreviations: ALND, axillary lymph node dissection; SLNB, sentinel lymph node biopsy.

^a^ There were 1317 facilities in total.

### Lymph Nodes Examined

**Figure 1.**
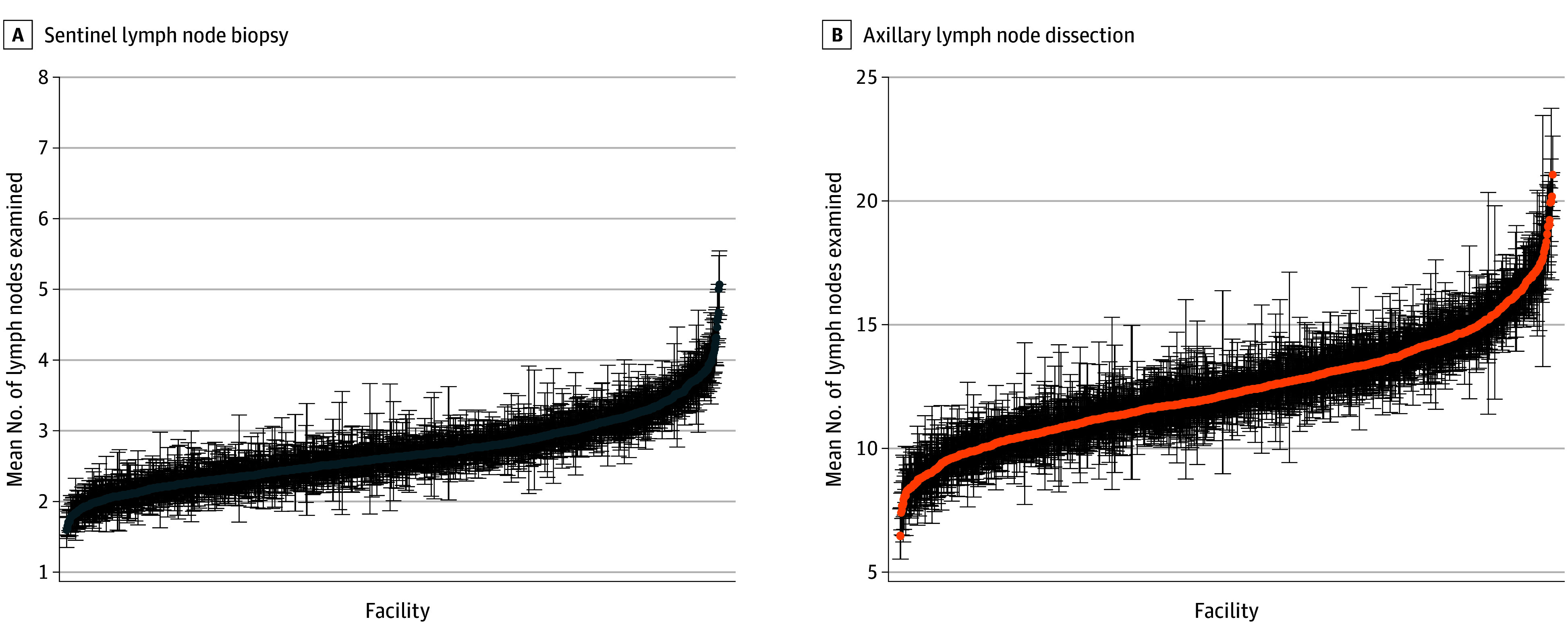
Reliability-Adjusted Estimates of Mean Lymph Nodes Examined During Axillary Surgery by Facility Error bars indicate 95% CIs.

Facility-level lymph node yield is summarized in Table 2. The median number of lymph nodes removed during SLNB was 2.6 (IQR, 2.3-3.0) and ranged from 1 to 6 across facilities. During ALND, a median of 12.2 (IQR, 10.9-13.6) lymph nodes were removed, ranging from 6 to 22 across facilities. Across the 1317 facilities, 163 (12.4%) performing ALND removed a mean of fewer than 10 lymph nodes for ALND. Median lymph node yield for ALND in those facilities was 9.3 (IQR, 8.9-9.8), ranging from 6.5 to 10.0. Facility-level variation in mean lymph node yield is displayed in Figure 1.

**Table 2.** Facility-Level Variation in Lymph Node Yield and Nodal Positivity Rates During Axillary Surgery

Procedure	Mean (95% CI)	Median (IQR)	Range
Lymph node yields (estimates)			
SLNB^a^	2.7 (2.7-2.7)	2.6 (2.3-3.0)	1-6
ALND^b^	12.4 (12.3-12.5)	12.2 (10.9-13.6)	6-22
Nodal positivity rates, %			
SLNB, ≥1 positive nodes^a^	12.5 (12.3-12.6)	12.2 (11.0-13.7)	6-21
ALND, ≥1 positive nodes^b^	66.8 (66.2-67.4)	67.5 (59.5-75.2)	32-94
ALND, ≥4 positive nodes^b^	30.8 (30.4-31.1)	30.5 (26.5-35.0)	11-54

Abbreviations: ALND, axillary lymph node dissection; SLNB, sentinel lymph node biopsy.

^a^ Lymph node yield obtained from 2018 to 2020.

^b^ Lymph node yield obtained from 2012 to 2020.

### Nodal Positivity Rate

**Figure 2.**
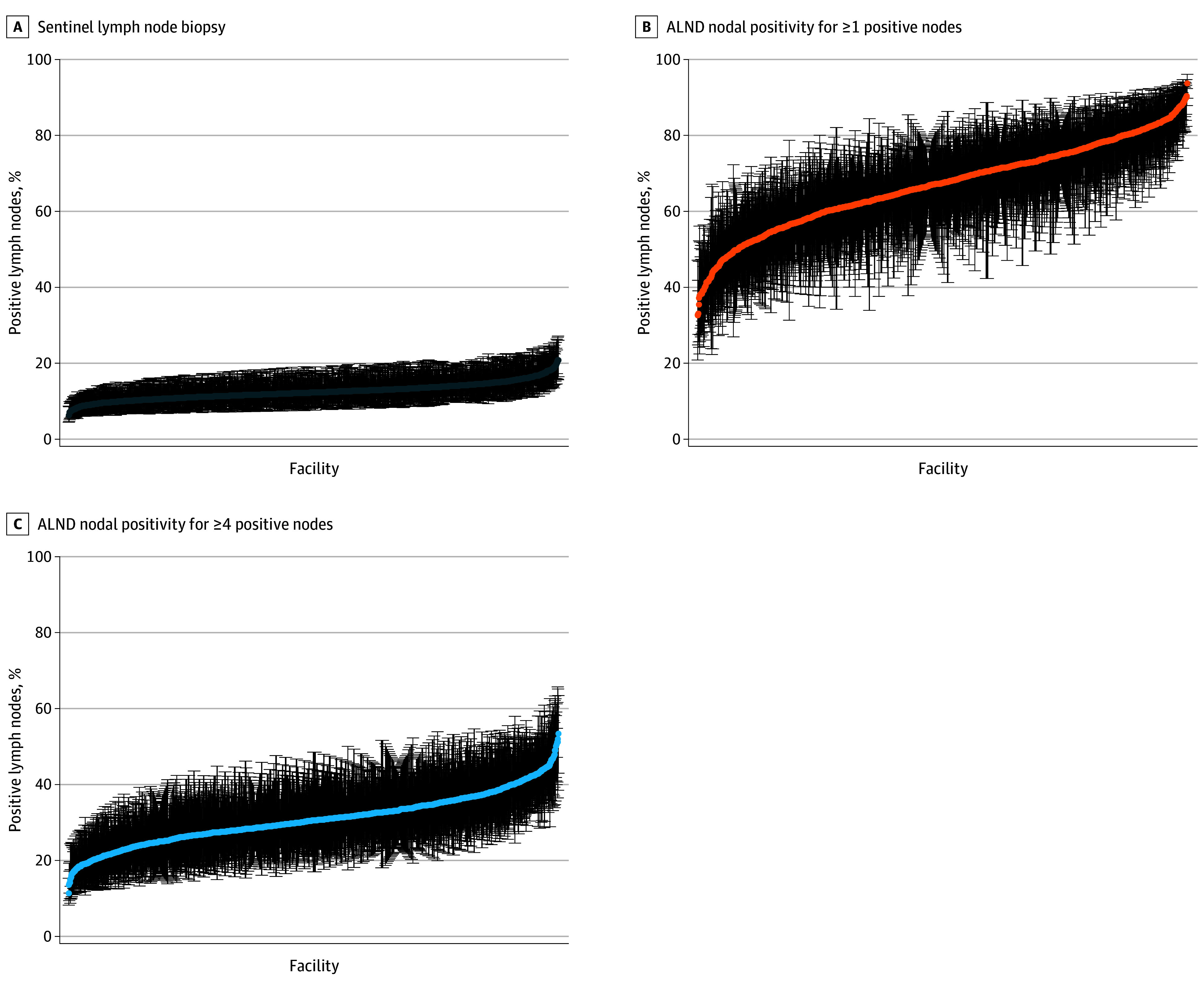
Reliability-Adjusted Rates of Nodal Positivity During Axillary Surgery by Facility ALND indicates axillary lymph node dissection. Error bars indicate 95% CIs.

The median nodal positivity rate (≥1 positive nodes) was 12.2% (IQR, 11.0%-13.7%) by facility for patients undergoing SLNB (Table 2). There was a 3-fold variation in the nodal positivity rate across facilities, with rates ranging from 6% to 21%. Patients undergoing ALND had a median nodal positivity rate of 67.5% (IQR, 59.5%-75.2%) for obtaining 1 or more positive nodes and 30.5% (IQR, 26.5%-35.0%) for obtaining 4 or more positive nodes. There was an almost 3-fold variation in the nodal positivity rate across facilities for obtaining 1 or more positive nodes, with rates ranging from 32% to 94%, and an almost 5-fold variation in the nodal positivity rate across facilities for obtaining 4 or more positive nodes, with rates ranging from 11% to 54%. Facility-level variation in nodal positivity rates for each axillary surgery is displayed in Figure 2.

### Correlation Between Lymph Nodes Examined and Nodal Positivity Rate by Facility

**Figure 3.**
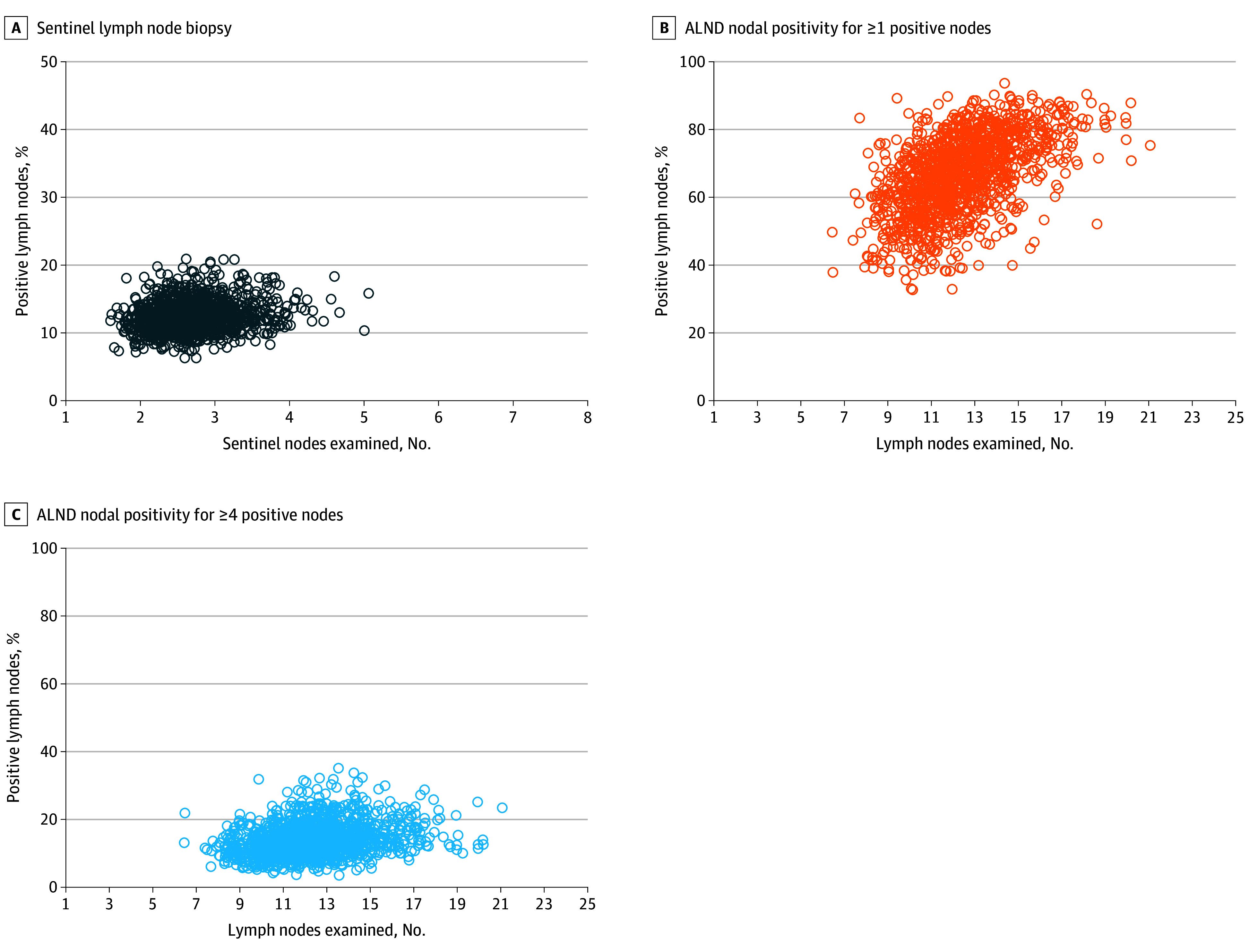
Scatterplots of Reliability-Adjusted Mean Lymph Nodes Examined and Nodal Positivity Rates During Axillary Surgery for Facilities ALND indicates axillary lymph node dissection.

To test the hypothesis that facilities examining more lymph nodes on average had higher nodal positivity, we performed correlations for each axillary surgery. The correlation between mean number of lymph nodes examined by facility and the rate of nodal positivity by facility for SLNB was weakly correlated (Spearman correlation coefficient, 0.17; *P* < .001) (Figure 3). Conversely, there was a strong correlation between mean number of lymph nodes examined by facility and the rate of 1 or more positive nodes (Spearman correlation coefficient, 0.54; *P* < .001) and 4 or more positive nodes (Spearman correlation coefficient, 0.53; *P* < .001) for ALND (Figure 3).

## Discussion

To our knowledge, this study is the first to examine the potential association of the ACS operative standards program with oncologic outcomes by examining facility-level variation in lymph node yield and nodal positivity rates for SLNB and ALND for patients undergoing surgery for breast cancer. These results help to identify the potential association with oncologic outcomes of implementing the ACS operative standards for breast cancer (standards 5.3 and 5.4), and we report several important findings. First, we have demonstrated facility-level variations in nodal yield for both SLNB and ALND, observing more variation for ALND. Second, we have observed a minimal correlation between facility-level lymph node yield and nodal positivity rates for SLNB, suggesting a possible ceiling effect with little potential association for further standardization. Conversely, we demonstrated a strong correlation between lymph node yield for ALND and identifying N2 disease (≥4 positive nodes), suggesting a larger potential association of technical standardization with oncologic outcomes for this procedure.

Sentinel lymph node biopsy is performed at high volumes across most hospitals, as demonstrated in our study. Although we found some facility-level variation in lymph node yield for SLNB, we found a weak correlation with facility-level nodal positivity rates. The role of axillary surgery in guiding adjuvant therapy recommendations is decreasing such that SLNB can be omitted for several subsets of women.^21,22^ Altogether, our findings suggest that a possible ceiling effect for the technical quality of SLNB has been reached, and further optimization may not lead to a clinically meaningful change in oncologic outcomes.

In contrast, we demonstrated more facility-level variation in lymph node yield for ALND, and this variation correlated with a greater likelihood of detecting 4 or more positive lymph nodes, a nodal stage that often triggers additional adjuvant therapy recommendations. Axillary lymph node dissection is a low-volume procedure for most surgeons and facilities in our study, and volume has dramatically decreased with the introduction of SLNB and clinical trials, such as the AMAROS (European Organisation for Research and Treatment of Cancer 10981-22023) and ACOSOG Z0011 (American College of Surgeons Oncology Group Z0011) trials, showing the safety of omitting completion ALND for patients with limited nodal disease.^23,24^ The diminishing indications for ALND have been shown to affect trainees, who are now graduating with less ALND experience.^25,26^

A possible association between volume and quality could lead to inaccurate staging or regional recurrence due to inadequate lymph node harvest during ALND. Several studies have shown an association between nodal yield during ALND and cancer outcomes. In one study, high nodal retrieval was associated with improved overall survival, presumably through greater use of adjuvant therapies, and a minimum yield of 20 lymph nodes was proposed as an adequate sample.^27^ The NCCN guidelines propose a much lower target, recommending retrieving at least 10 lymph nodes during ALND.^11^ Patient factors, such as obesity, are associated with variation in lymph node yield for an individual patient.^6,28,29,30,31^ In addition, obtaining fewer than 10 lymph nodes during ALND may be an adequate dissection for an individual patient. However, the number of facilities consistently not meeting this quality measure suggests a possible quality gap in surgical technique or pathologic examination. The facilities in our study consistently not achieving this metric may represent CoC sites with the most to benefit from implementation of the ACS operative standards. Alternatively, a few facilities had a very high median nodal yield during ALND. This could indicate an overly aggressive ALND targeting level III nodes, which would place patients at increased risk of lymphedema, or represent variation in how lymph nodes are pathologically counted or examined.^32,33^ Taken as a whole, our findings suggest that there is potential room for improvement in the technical quality of ALND at many CoC facilities, which could improve oncologic outcomes for patients with breast cancer. Implementation of the ACS operative standards will provide an opportunity to identify which factors play a role in lymph node sample yield, provide insight on how to address them to achieve an optimal outcome, evaluate the association of many of these factors with oncologic outcomes, and potentially address them. For example, the synoptic operative reporting requirement by the CoC offers a structured format for reporting the technical aspects of axillary surgery in prespecified terminology that can be extracted and analyzed.^34,35,36^

Our results demonstrated a large variation in nodal positivity rates (≥1 positive node) across facilities for ALND. As SLNB should be performed as initial staging for nearly all patients, the expected nodal positivity rates across facilities for ALND should approach 100%,^37,38^ with only a few selected scenarios where clinically node-negative disease may undergo ALND (eg, inflammatory breast cancer).^11,39^ Low rates of nodal positivity for ALND demonstrated in our study suggest a potential opportunity for de-escalation of upfront ALND in favor of SLNB and suggest a possible role for reporting the indication for ALND in the associated synoptic report.

This study provides a framework for guiding future efforts to improve technical quality in surgery. The ACS has proposed over 130 operative standards and selected 6 for implementation, 4 of which will be reported by surgeons using synoptic operative reports. This large and previously unprecedented attempt to standardize technical quality must be considered in the context of the administrative burden that quality metric implementation and reporting can have on surgeons and hospitals. Quality metric reporting has been shown to exceed 100 000 person-hours and $5 million per year for a single hospital, which can place an undue burden on poorly resourced hospitals.^40^ Adding to this reporting burden should be done only when there is strong evidence that the metrics and reporting improve patient outcomes. Understanding the existing variation in technical quality is the first step in identifying how the current operative standards may be used to improve patient outcomes and to help determine how to refine and prioritize future quality improvement initiatives so they have the greatest association with oncologic outcomes.

### Limitations

Several important limitations should be noted in our study. First, this was a retrospective analysis of the NCDB and is subject to missing or incorrect documentation of key variables, such as the type of axillary surgery performed. If misclassification of axillary surgery type occurred or information on lymph node yield was not provided, these could have altered the variation we observed in lymph node yield and subsequent nodal positivity. Second, the low volume of ALND procedures performed in this study could have limited the reliability of some estimates obtained. The nodal yield for low-volume hospitals could be more subject to variation due to patient-level factors and case mix; however, as we included 8 years of data, these estimates likely reflect the best available. Third, the NCDB does not classify the use of targeted axillary dissection, which is becoming more common for patients with clinically node-positive disease receiving neoadjuvant chemotherapy.^41^ To address this limitation, we excluded patients receiving neoadjuvant chemotherapy. Fourth, we used short-term oncologic process outcomes, such as lymph node yield and nodal positivity rate, but did not directly evaluate patient-centered outcomes, such as recurrence and disease-specific survival. As recurrence data are not available in the NCDB, assessment of these longer-term outcomes is not possible outside of the NCDB Special Study mechanism.^42,43^ Future prospective studies evaluating these outcomes in association with lymph node yield are underway.

## Conclusions

In this cohort study of women undergoing axillary surgery for breast cancer, facility-level variation was observed in the nodal yield for SLNB and ALND, which could be addressed through technical standardization. The facility-level variation in SLNB was weakly correlated with nodal positivity, while it was strongly correlated with upstaging rates in ALND. This study’s findings suggest the ACS operative standards program could have mixed associations with short-term oncologic outcomes in breast cancer and overall provide a framework for future work in refining and prioritizing operative standards.

## Notes

Supplement 1. **eTable 1.** Axillary Surgery Case Volume **eTable 2.** Correlation Between Mean Lymph Node Yield and Nodal Positivity During Axillary Surgery **eTable 3.** Yearly SLNB Case Volume From 2018-2020 **eTable 4.** Yearly ALND Case Volume From 2012-2020 **eTable 5.** Yearly SLNB With Completion ALND Case Volume From 2012-2020

Supplement 2.
Data Sharing Statement
