# Supplementary material for: American College of Surgeons Operative Standards and Breast Cancer Outcomes
Source: JAMA Netw Open. 2024 Nov 20;7(11):e2446345. doi: 10.1001/jamanetworkopen.2024.46345 (PMC11579798; doi:10.1001/jamanetworkopen.2024.46345)
Supplement: Supplement 1. — eTable 1. Axillary Surgery Case Volume eTable 2. Correlation Between Mean Lymph Node Yield and Nodal Positivity During Axillary Surgery eTable 3. Yearly SLNB Case Volume From 2018-2020 eTable 4. Yearly ALND Case Volume From 2012-2020 eTable 5. Yearly SLNB With Completion ALND Case Volume From 2012-2020 [file jamanetwopen-e2446345-s001.pdf]

Supplementary Online Content

Taylor CD, Wang T, Baskin AS, et al. American College of Surgeons operative standards and breast cancer outcomes. *JAMA Netw Open.* 2024;7(11):e2446345. doi:10.1001/jamanetworkopen.2024.46345

eTable 1. Axillary Surgery Case Volume
eTable 2. Correlation Between Mean Lymph Node Yield and Nodal Positivity During Axillary Surgery
eTable 3. Yearly SLNB Case Volume From 2018-2020
eTable 4. Yearly ALND Case Volume From 2012-2020
eTable 5. Yearly SLNB With Completion ALND Case Volume From 2012-2020

This supplementary material has been provided by the authors to give readers additional information about their work.

© 2024 Taylor CD et al. *JAMA Network Open.*

eTable 1. Axillary Surgery Case Volume

Procedure	Mean (95% CI)	Median (IQR)	Range
SLNB[a]	84.1 (79.1-89.0)	57.3 (28.0-110.0)	1-1361
ALND[b]	8.8 (8.3-9.3)	6.4 (3.6-11.0)	1-118
SLNB with Completion ALND[b]	16.3 (15.2-17.3)	10.7 (5.7-20.3)	1-280

Abbreviations: SLNB, Sentinel Lymph Node Biopsy; ALND, Axillary Lymph Node Dissection
[a] Case volume obtained from 2018 - 2020
[b] Case volume obtained from 2012 – 2020

© 2024 Taylor CD et al. *JAMA Network Open.*

eTable 2. Correlation Between Mean Lymph Node Yield and Nodal Positivity During Axillary Surgery

Procedure	Outcome 1	Outcome 2	Correlation[c]
SLNB[a]	≥1 positive nodes	Mean lymph nodes	0.17
ALND[b]	≥1 positive nodes	Mean lymph nodes	0.54
ALND[b]	≥4 positive nodes	Mean lymph nodes	0.53

Abbreviations: SLNB, Sentinel Lymph Node Biopsy; ALND, Axillary Lymph Node Dissection
[a] Obtained from 2018 - 2020
[b] Obtained from 2012 - 2020
[c] Spearman correlation coefficient

© 2024 Taylor CD et al. *JAMA Network Open.*

eTable 3. Yearly SLNB Case Volume From 2018-2020

Year	Mean (95% CI)	Median (IQR)	Range
2018	84.4 (79.3-89.5)	57 (28-109)	1-1523
2019	92.4 (86.9-97.9)	63 (32-121)	1-1597
2020	78.5 (73.9-83.1)	55 (25-106)	1-961

Abbreviation: SLNB, Sentinel Lymph Node Biopsy

© 2024 Taylor CD et al. *JAMA Network Open.*

eTable 4. Yearly ALND Case Volume From 2012-2020

Year	Mean (95% CI)	Median (IQR)	Range
2012	12.7 (11.9-13.5)	9 (5-16)	1-213
2013	12.6 (11.7-13.4)	9 (4-16)	1-253
2014	11.1 (10.4-11.8)	8 (4-14)	1-217
2015	10.2 (9.6-10.8)	7 (3-13)	1-113
2016	9.4 (8.8-9.9)	6 (3-12)	1-81
2017	8.9 (8.4-9.5)	6 (3-12)	1-87
2018	8.2 (7.7-8.7)	5 (3-10)	1-115
2019	6.0 (5.6-6.4)	4 (2-8)	1-73
2020	4.9 (4.6-5.2)	3 (2-6)	1-64

Abbreviation: ALND, Axillary Lymph Node Dissection

© 2024 Taylor CD et al. *JAMA Network Open.*

eTable 5. Yearly SLNB With Completion ALND Case Volume From 2012-2020

Year	Mean (95% CI)	Median (IQR)	Range
2012	21.4 (19.9-22.8)	14 (7-27)	1-272
2013	21.3 (19.9-22.7)	14 (6-27)	1-315
2014	19.7 (18.4-21.1)	13 (6-25)	1-326
2015	18.9 (17.6-20.3)	12 (6-24)	1-339
2016	18.0 (16.6-19.3)	11 (5-21)	1-349
2017	16.7 (15.5-18.0)	10 (5-21)	1-336
2018	14.0 (12.9-15.1)	8 (4-17)	1-279
2019	13.5 (12.5-14.5)	9 (4-17)	1-277
2020	11.2 (10.4-12.0)	7 (3-14)	1-151

Abbreviations: SLNB, Sentinel Lymph Node Biopsy; ALND, Axillary Lymph Node Dissection

© 2024 Taylor CD et al. *JAMA Network Open.*
