# Supplementary material for: American College of Surgeons Operative Standards and Breast Cancer Outcomes
Source: JAMA Netw Open. 2024 Nov 20;7(11):e2446345. doi: 10.1001/jamanetworkopen.2024.46345 (PMC11579798; doi:10.1001/jamanetworkopen.2024.46345)
Supplement: Supplement 2. — Data Sharing Statement [file jamanetwopen-e2446345-s002.pdf]

Data Sharing Statement

Taylor. American College of Surgeons Operative Standards and Breast Cancer Outcomes. *JAMA Netw Open*. Published November 20, 2024. doi:10.1001/jamanetworkopen.2024.46345

Data
Data available: No

Additional Information
Explanation for why data not available: We used data from the National Cancer Database which is controlled by the ACS and available upon request and application submission. Methods for our analysis would be available upon request.
